# Oxygen Requirement in Overweight/Obese Kidney Transplant Recipients with COVID-19: An Observational Cohort Study

**DOI:** 10.3390/diagnostics13132168

**Published:** 2023-06-26

**Authors:** Alexandre Veronese-Araújo, Débora D. de Lucena, Isabella Aguiar-Brito, Luís Gustavo Modelli de Andrade, Marina P. Cristelli, Hélio Tedesco-Silva, José O. Medina-Pestana, Érika B. Rangel

**Affiliations:** 1 Department of Medicine, Nephrology Division, Federal University of São Paulo, São Paulo 04038-031, SP, Brazil; 2 Hospital do Rim, São Paulo 04038-002, SP, Brazil; 3 Department of Internal Medicine, Botucatu Medical School, University of São Paulo State, Botucatu 18618-687, SP, Brazil; 4 Hospital Israelita Albert Einstein, São Paulo 05652-900, SP, Brazil

**Keywords:** overweight, obesity, kidney transplant, COVID-19, outcomes

## Abstract

Introduction: Obesity is one of the components of the cardiometabolic syndrome that contributes to COVID-19 progression and mortality. Immunosuppressed individuals are at greater risk of the COVID-19 burden. Therefore, we sought to investigate the impact of the combination of overweight/obesity and kidney transplant on oxygen (O_2_) requirements in the COVID-19 setting. Methods: Retrospective analysis of 284 kidney transplant recipients (KTRs) from March/2020 to August/2020 in a single center. We investigated the risk factors associated with O_2_ requirements in overweight/obese KTRs. Results: Overall, 65.1% had a BMI (body mass index) ≥ 25 kg/m^2^, 52.4% were male, the mean age was 53.3 ± 11 years old, 78.4% had hypertension, and 41.1% had diabetes mellitus. BMI was an independent risk factor for O_2_ requirements (OR = 1.07, *p* = 0.02) alongside age, lymphopenia, and hyponatremia. When overweight/obese KTRs were older, smokers, they presented higher levels of lactate dehydrogenase (LDH), and lower levels of estimated glomerular filtration rate (eGFR), lymphocytes, and sodium at admission, and they needed O_2_ more often. Conclusion: Being overweight/obese is associated with greater O_2_ requirements in KTRs, in particular in older people and smokers, with worse kidney allograft functions, more inflammation, and lower sodium levels. Therefore, the early identification of factors that predict a worse outcome in overweight/obese KTRs affected by COVID-19 contributes to risk stratification and therapeutic decisions.

## 1. Introduction

Solid-organ transplant (SOT) individuals hospitalized for COVID-19 present high rates of mortality, which are associated with age, sex, and pre-existing comorbidities such as congestive heart failure, hypertension, chronic lung disease, diabetes mellitus, and obesity [1,2,3].

When compared to non-SOT individuals, SOT individuals with COVID-19 demonstrated not only an increase in odds of mortality (1.94), but also higher rates of transferring to the ICU (1.46), receiving invasive mechanical ventilation (2.34), and developing an acute kidney injury (2.41) [4]. Conversely, a propensity score-matched analysis pointed out similar rates of COVID-19 severity in SOT and non-SOT patients [5], as well as in diabetics and non-diabetic kidney transplant recipients (KTRs) with COVID-19 [6].

Despite the growing literature focusing on the prognosis of COVID-19 in transplant recipients, data on selected high-risk clinical populations that deserve special consideration, such as immunocompromised individuals with a history of overweight and obesity, remain undetermined.

Obesity is frequently associated with other comorbidities, which puts patients at greater risk of COVID-19 progression and mortality [7]. Additionally, dysfunctional adipose tissue in obesity plays an important role in the secretion of pro-inflammatory cytokines and SARS-CoV-2 pathogenicity, leading to an increase in the COVID-19 burden [8].

To note, identifying the impact of being overweight/obesity in a high-risk group of an immunosuppressed population is of paramount importance for defining therapeutic decisions, patient flow management, and the allocation of resources in the COVID-19 setting [9]. Therefore, we sought to investigate the risk factors associated with oxygen requirements in overweight/obese KTRs with COVID-19.

## 2. Patients and Methods

### 2.1. Study Design and Setting

A cohort, cross-sectional, observational, and descriptive study was conducted at Hospital do Rim, São Paulo, SP, Brazil. The medical records of patients who were either hospitalized or non-hospitalized with the diagnosis of COVID-19 during the study period of March to August 2020 were assessed, corresponding to the first wave of COVID-19 in Brazil. We only included patients in whom SARS-CoV-2 was detected by nasopharyngeal swab RT- PCR (reverse transcriptase polymerase chain reaction). The population at risk included 11,875 KTRs. Of the 590 KTRs who became ill, 284 were included in the study. We excluded 306 KTRs due to the following reasons: double transplant (*n* = 6), kidney allograft was lost in the period before COVID-19 (*n* = 4), delayed graft function at the time of diagnosis of COVID-19 (*n* = 4), no use of immunosuppressive drugs due to cancer treatment (*n* = 1), <18 years-old (*n* = 1), and missing data due to admission to other services (*n* = 290).

A standardized data collection form was developed to retrospectively retrieve relevant information from medical records. Data were collected regarding patient demographics and laboratory parameters on admission with COVID-19 symptoms. The last patient was included in the study on 30 August 2020. The Ethics and Research Committee of the Federal University of São Paulo (CAEE 35311020.9.0000.8098) approved the study. Informed consent was obtained from all patients, whereas a waiver was granted for patients who died in other hospitals.

Patient demographics include age, sex, race, body mass index (BMI), type of donor, time of transplant, as well as the presence of comorbidities (smoking, hypertension, DM, chronic obstructive pulmonary disease (COPD), heart disease, liver disease, and autoimmune disease) were collected. We also evaluated the symptoms on admission.

DM was defined according to the use of insulin and/or oral antidiabetics, hypertension whether individuals were on anti-hypertensive drugs, liver disease whether hepatitis B or C were diagnosed, and heart disease whether heart failure and/or coronary artery disease were present. BMI analysis was performed using the World Health Organization criteria: < 25 kg/m^2^ considered normal, ≥ 25–29.9 kg/m^2^ considered overweight, and ≥ 30 kg/m^2^ considered obesity.

### 2.2. Laboratory Testing

On admission, we evaluated in-hospital laboratory data: lymphocytes, serum creatinine, glycemia, aspartate aminotransferase (AST), alanine aminotransferase (ALT), D-dimer, lactate dehydrogenase (LDH), and C-reactive protein (CRP). As for laboratory data before admission, we collected baseline creatinine (meaning the last three measurements), fasting blood glucose (FBG; last measurement within 6 months), and glycated hemoglobin (HbA1c; last measurement within the 1 year).

The estimated glomerular filtration rate (eGFR) was calculated using the formula defined in the CKD-EPI (Chronic Kidney Disease Epidemiology Collaboration) study: 175 × serum creatinine and 1.154 × age and 0.203 × 1.212 (if black) × 0.742 (if woman), and was expressed in mL/min/1.73 m^2^ of the body surface.

### 2.3. Statistical Analyses

The primary aim was to investigate supplemental oxygen (O_2_) requirement in overweight/obese compared to lean KTRs with COVID-19. The secondary aim was to investigate the risk factors associated with O_2_ in overweight/obese KTRs. Therefore, to investigate whether BMI was an independent risk factor for COVID-19 severity, we performed univariate analyses of demographic and laboratory data, and when *p*-value was ≤ 0.1, the variables were entered simultaneously into a binary logistic regression model. We also evaluated other outcomes, such as death, transfer to intensive care unit (ICU), acute kidney injury (AKI) classified according to KDIGO guidelines, need for hemodialysis (HD), supplemental O_2_, and invasive mechanical ventilation (IMV), as previously described for COVID-19 progression classification [10]. Oxygen requirement was defined as any use of oxygen, including nasal prongs, masks, and non-invasive ventilation or high-flow, in particular when patients presented a SatO_2_ < 94% on room air and dyspnea. The results were expressed as odds ratios (ORs) with a confidence interval (CI) of 95%.

We estimated that O_2_ requirement in KTRs with COVID-19 was 15% [11], whereas in obese/overweight people it was 30% [12]. Using an alpha of 0.05 and a beta of 0.2 in two-sided approach based on power calculation for two proportions, we retrieved a total number of 236 patients. For the need of O_2_, we also used the Multiple Imputation by Chained Equations (MICE) for handling missing values in the multivariate logistic regression. The basic idea behind MICE is to use observed values from other variables in the dataset to impute the missing values. This is performed by creating a regression model for each variable with missing values, where the other variables in the dataset are used as predictors. The regression models are then used to impute the missing values for each variable, one at a time. This process is repeated for a set number of iterations until the imputed values converge to stable values. The MICE generated plausible numbers derived from distributions and relationships among observed variables in the data set and followed three steps: (1) generating replacement values for missing data and repeating this procedure 10 times; (2) analyzing the 10 imputed data sets; and (3) pooling the results according to Rubin Rules. MICE is a powerful tool for dealing with missing data, as it provides a framework for making valid inferences in the presence of missing values.

BMI was also evaluated as a continuous variable in a model of linear regression using demographic and laboratory data as well as those outcomes.

Data were described as mean ± standard deviation or median and interquartile range, as indicated. Frequencies and percentages were reported for qualitative data.

Receiver Operating Characteristic (ROC) curves were used to identify the laboratory parameters associated with COVID-19-related outcomes. To calculate the LDH, lymphocytes, and sodium cut-off values with better sensitivity and specificity for outcomes, we used the Youden index.

We analyzed the data using IBM^®^ SPSS (Statistical Product and Services Solutions, version 18.0, SPSS Inc, Chicago, IL, USA). A *p*-value of < 0.05 was considered significant for all data analyses.

## 3. Results

In our cohort, from 284 KTRs who were evaluated, 185 (65.1%) had a BMI ≥ 25 kg/m^2^. The majority was overweight (64.9%, *n* = 120), whereas the obese individuals were distributed as follows: 30–34.9 kg/m^2^ (27%, *n* = 50), 35–39.9 kg/m^2^ (4.9%, *n* = 9), and ≥ 40 kg/m^2^ (3.2%, *n* = 6).

By univariate analysis, BMI was an independent risk factor for the need for O_2_ (OR = 1.06, 95% CI 1.00–1.11, *p* = 0.03, Table 1), in particular when greater than 25 kg/m^2^ (OR = 1.68, 95% CI 1.03–2.75, *p* = 0.04, Table 1) in KTRs with COVID-19.

**Figure 1:**
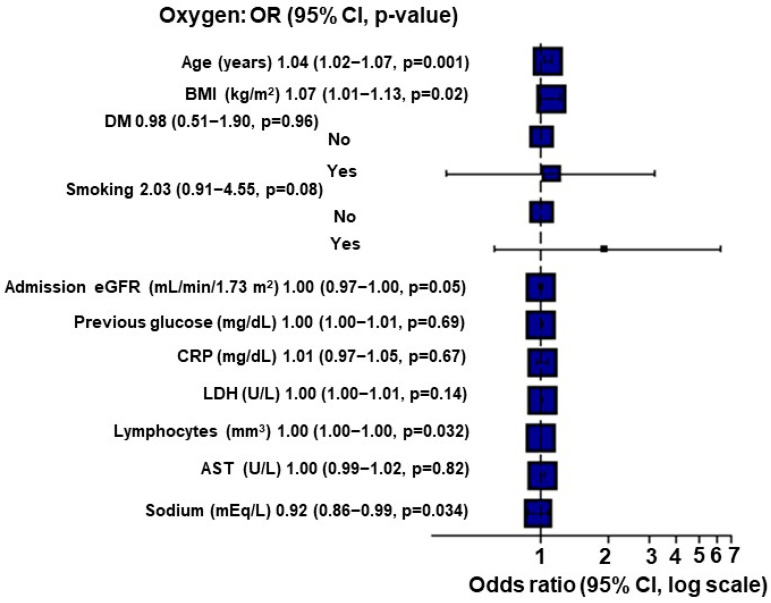
Multivariate analysis for O_2_ requirement using Multiple Imputation by Chained Equations (MICE) in kidney transplant recipients (*n* = 284).

When applying Multiple Imputation by Chained Equations (MICE), for each point of increase in the BMI, there was a 7% increase in the O_2_ requirement (*p* = 0.02; Figure 1).

However, BMI was not an independent risk factor for other COVID-19-related outcomes, such as mortality (Table S1), transfer to ICU (Table S2), the need for IMV (Table S3), AKI development (Table S4), and HD requirement (Table S5) in these patients.

Clinical and epidemiological characteristics and laboratorial data of overweight/obese kidney transplant recipients are described in Table 2 and Table 3, respectively.

Next, we evaluated the risk factors associated with the need for supplemental O_2_ in overweight/obese KTRs. By univariate analysis, we found that age (55.7 ± 10.6 vs. 49.9 ± 10.8 years-old, *p* = 0.001), smoking (29.6% vs. 14.3%, *p* = 0.02), lower eGFR on admission (34.3 ± 20.3 vs. 42.5 ± 20.4 mL/min/1.73 m^2^, *p* = 0.01), higher levels of LDH (365.9 ± 190.4 vs. 300.0 ± 172.7 U/L, *p* = 0.049), lower lymphocytes levels (821.2 ± 549.3 vs. 1157.6 ± 832.9/mm^3^, *p* = 0.003), and hyponatremia (134.5 ± 5.6 vs. 136.1 ± 3.4 mEq/L, *p =* 0.04) contributed to COVID-19 severity. In the multivariate analysis, only age (*p* = 0.001) was statistically significant for the O_2_ requirements (Table 4).

**Figure 2:**
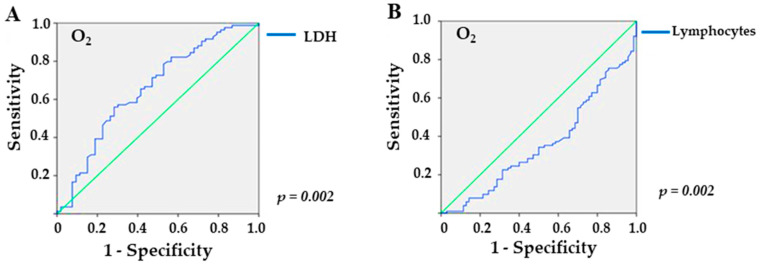
Sensitivity and specificity of laboratory markers in overweight/obese KTRs with COVID-19 and need for supplemental O_2_: (**A**) LDH (lactate dehydrogenase; *p* = 0.002), and (**B**) lymphocytes (*p* = 0.002), the green line is the reference line.

In the analysis of the ROC curve, referring to the overweight/obesity group, LDH provided an area under the curve (AUC) of 0.656 (*p* = 0.002), with a sensitivity of 56% and a specificity of 71.7% for values higher than 309.5 U/L (Figure 2A) in patients who required O_2_ supplementation. In addition, lymphocyte levels yielded an AUC of 0.652 (*p* = 0.002), with a sensitivity of 60.8% and a specificity of 65.7% for values lower than 743.5 mm^3^ (Figure 2B). For sodium, the ROC curve yielded no significant value (*p* = 0.1).

To further substantiate our findings, we also performed a linear regression model using BMI as a continuous and dependent variable (Tables S6 and S7). BMI was also a risk factor for O_2_ requirement (OR = 1.27, *p* = 0.03; Table S7) in KTRs.

## 4. Discussion

Our study showed that overweight/obese KTRs affected by COVID-19 have a greater susceptibility to the need for O_2_. The risk factors associated with this outcome included demographic variables, such as older age and smoking, and laboratory parameters, such as lower eGFR, higher LDH levels, lymphopenia, and hyponatremia on admission.

The association between obesity and COVID-19 severity may be explained by several mechanisms. Firstly, adipose tissue (AT) expresses the angiotensin-2-converting enzyme (ACE2) receptor [13], which is used by SARS-CoV-2 to enter host cells [14]. Therefore, AT may be a reservoir for SARS-CoV-2 multiplication, which ultimately leads to an increase in the COVID-19 burden. In addition, ACE2 gene expression was found to be higher in both subcutaneous and visceral AT when compared to lung cells in obese patients [15,16], as well as in the soluble form of ACE2 in patients with a metabolic syndrome [17].

Nonetheless, SARS-CoV-2 may not only promote direct cell toxicity but also dysregulate the immune system [18], which promotes endothelial cell damage associated with thromboinflammation [19] and an imbalance of the renin–angiotensin–aldosterone and kallikrein–kinin systems [14].

Secondly, another characteristic of excess AT that is linked to the inflammatory imbalance of obesity is the abnormality in the production of adipokines, such as the upregulation of proinflammatory adipokines (leptin) and the downregulation of anti-inflammatory adipokines (adiponectin), which aggravates cerebrovascular and cardiovascular diseases and insulin resistance [20]. Moreover, a low concentration of adiponectin in obese individuals is associated with greater levels of IL-6, TNF-α, and chemokines, which may contribute to the susceptibility to viral lung infection [21], and ultimately, to O_2_ requirements, as observed in our study.

Thirdly, the higher the grade of obesity, the lower the levels of the PaO_2_/FIO_2_ ratio were observed, which may be attributable to a failure in the ventilation capacity due to mechanical impairment and the burden of COVID-19 [22,23,24,25].

Finally, the underlying comorbidities found in obese individuals such as heart, kidney, and pancreatic diseases may also be associated with the dysregulation of the ACE/ACE2 axis in these disease-related tissues [16,26]. Glucocorticoid use in KTRs can also contribute to obesity [27], which puts KTRs at great risk of COVID-19 severity. Likewise, smoking upregulates ACE2 expression within the lungs [28], which explains the higher need for O_2_ in our population of overweight/obese KTRs who were smokers. Other obesity-related comorbidities, such as obstructive apnea syndrome and obesity hypoventilation syndrome, may also play a role in O_2_ requirements [23], yet they are not evaluated in our population.

When we verified other risk factors for the need for O_2_ in overweight/obese KTRs, we observed that age was associated with COVID-19 severity. Age is associated not only with a higher number of underlying comorbidities [29] but also with the aging of the immune system [30]. In that setting, the vicious circle of cellular senescence and immune cell dysfunction leads to inflammaging and immunosenescence, and vice versa.

Thus, immune system dysregulation leads to lymphopenia, a hallmark of COVID-19 severity [31], including in transplanted individuals [1,3,32], as we also observed in our overweight/obese population who required O_2_ supplementation. Immunosuppressive regimens and in particular, anti-proliferative drugs, aggravate SARS-CoV-2 infection and are usually withdrawn in KTRs diagnosed with COVID-19 [32].

To note, higher levels of LDH are also a hallmark of COVID-19 severity and tissue damage in both transplanted [32,33] and non-transplanted individuals [34], as we also documented in overweight/obese KTRs who required O_2_ supplementation. Therefore, LDH is also a helpful tool for risk-stratifying the COVID-19 burden in the transplant setting.

Other risk factors for O_2_ supplementation comprised lower kidney function and lower sodium levels in our population of people who were overweight. Chronic kidney disease (CKD) is associated with worse outcomes in COVID-19 [35] due to higher levels of thromboinflammation markers (lymphopenia, neutrophilia, thrombocytopenia, imbalance of thrombosis/fibrinolysis, increased levels of procalcitonin, and LDH) [36]. Acute kidney injury superimposed on CKD occurs in either transplanted [32] or non-transplanted individuals [36] and exhibits a temporal association with respiratory failure [37].

In addition, hyponatremia has been reported in 9.1 to 51.8% of COVID-19 patients and includes several mechanisms, such as a syndrome of inappropriate antidiuretic hormone secretion, sodium loss due to diarrhea, reduced sodium intake, or use of diuretic therapy, as reviewed elsewhere [38], and is a prognostic factor for COVID-19 progression [39]. In KTRs, the impact of sodium levels on the COVID-19 burden warrants further investigation in larger cohorts.

Given the impact of weight gain post-transplants [40], our findings encourage the establishment of pharmacologic and non-pharmacologic approaches, such as lifestyle modification, to mitigate the impact of increased BMI, a modifiable variable, on COVID-19 severity.

While the limited sample size warrants a cautious interpretation, our findings highlight the importance of assessing the contribution of adipose tissue on COVID-19 severity in a larger cohort in the transplant setting. Importantly, we did not find obesity-related comorbidities, such as DM, hypertension, and cardiac disease as predictors of O_2_ requirement, which may be explained by the fact that we analyzed a small sample and these diseases were highly prevalent in KTRs in both lean and overweight/obese individuals (36.4% vs. 41.1%, 69.4% vs. 78.4%, and 8.1% vs. 13%, respectively). In the overweight/obese group, these comorbidities were also equally found in those who needed O_2_ and those who did not. To note, the prevalence of these obesity-related outcomes was higher when compared to the general population, which may point to a different impact of obesity on an immunosuppressive burden. The fact that all data were retrospectively collected should also be of note. Therefore, the impact of longitudinal analyses of laboratory parameters and the evaluation of vaccination on COVID-19 outcomes in KTRs who are overweight/obese should be pursued during the pandemic.

In conclusion, age, BMI, lymphopenia, and hyponatremia are associated with O_2_ requirements in KTRs. Overweight/obesity is a risk factor for COVID-19 severity, requiring increased attention to preventive measures in susceptible KTRs, in particular in older smokers individuals with altered laboratory parameters.

## Figures and Tables

**Table 1:** Risk factors for the use of supplemental oxygen (O_2_) on admission in kidney transplant recipients (*n* = 284).

Variables	O_2_ (*n* = 153, 53.9%)	No O_2_ (*n* = 131, 46.1%)	Univariate Analysis
Age (years)	55.4 ± 12.0	49.0 ± 11.6	1.05 (1.03–1.07, *p =* 0.0001)
Male (*n*, %)	82 (53.6)	78 (51.0)	0.79 (0.49–1.26, *p* = 0.31)
Race (*n*, %) White Black/brown	96 (62.7) 57 (37.3)	77 (50.7) 54 (35.3)	1.18 (0.73–1.90, *p* = 0.50)
Transplant time (months)	92.1 ± 71.4	94.6 ± 72.5	1.00 (1.00–1.00, *p* = 0.77)
Donor type (*n*, %) Alive Deceased	39 (25.5) 114 (74.5)	41 (46.4) 90 (58.8)	1.33 (0.79–2.24, *p* = 0.28)
BMI (kg/m^2^)	27.5 ± 4.9	26.2 ± 4.8	1.06 (1.00–1.11, *p* = 0.03)
BMI ≥ 25 (*n*, %)	108 (70.6)	77 (50.7)	1.68 (1.03–2.75, *p* = 0.04)
BMI ≥ 30 (*n*, %)	41 (26.8)	24 (15.7)	1.63 (0.92–2.88, *p* = 0.09)
Hypertension (*n*, %)	114 (74.5)	100 (65.4)	0.91 (0.53–1.56, *p* = 0.72)
Diabetes mellitus (*n*, %)	68 (44.4)	44 (28.8)	1.58 (0.98–2.56, *p* = 0.06)
COPD (*n*, %)	7 (4.6)	2 (1.3)	3.09 (0.63–15.15, *p* = 0.16)
Heart disease (*n*, %)	19 (12.4)	13 (8.5)	1.29 (0.61–2.72, *p* = 0.51)
Neoplasia (*n*, %)	14 (9.2)	7 (4.6)	1.78 (0.70–4.56, *p* = 0.23)
Liver disease (*n*, %)	8 (5.2)	1 (0.7)	7.17 (0.88–58.12, *p* = 0.06)
Autoimmune disease (*n*, %)	6 (3.9)	2 (1.3)	2.63 (0.52–13.27, *p* = 0.24)
Smoking (*n*, %)	41 (26.8)	18 (11.8)	2.12 (1.13–3.99, *p* = 0.02)
Laboratory data			
Basal eGFR	47.5 ± 23.6	50.2 ± 24.5	1.00 (0.99–1.00, *p* = 0.34)
Admission eGFR	34.4 ± 21.7	40.2 ± 21.8	0.99 (0.98–01.00, *p* = 0.03)
Previous glucose (mg/dL)	127.2 ± 65.7	112.2 ± 66.7	1.00 (1.00–1.01, *p* = 0.07)
Admission glucose (mg/dL)	177.9 ± 105.9	167.0 ± 102.2	1.00 (1.00–1.00, *p* = 0.61)
Previous Hb1Ac (%)	7.0 ± 2.0	6.7 ± 2.1	1.09 (0.95–1.26, *p* = 0.20)
CRP (mg/dL)	8.7 [4.0;15.2]	3.3 [1.0;9.6]	1.06 (1.02–1.09, *p* = 0.001)
LDH (U/L)	341.0 [241.5;430.5]	239.0 [182.8;308.0]	1.00 (1.00–1.01, *p* = 0.0001)
Lymphocytes (mm^3^)	658.5 [429.0;997.0]	909.5 [584.0;1314.0]	1.00 (1.00–1.00, *p* = 0.004)
D-dimer (µg/L)	1.2 [0.6;2.4]	1.2 [0.5;2.1]	1.03 (0.96–1.11, *p* = 0.37)
AST (U/L)	31.0 [20.0;44.0]	28.0 [22.5;37.5]	1.01 (1.00–1.02, *p* = 0.06)
ALT (U/L)	21.0 [13.5;33.0]	21.0 [16.0;29.3]	1.01 (1.00–1.02, *p* = 0.22)
Sodium (mEq/L)	134.2 ± 5.8	135.9 ± 3.7	0.93 (0.88–0.98, *p* = 0.01)

BMI: body mass index in kg/m^2^; eGFR: estimated glomerular rate, in mL/min/1.73 m^2^; Hb1Ac: glycated hemoglobin; CRP: C-reactive protein; LDH: lactate dehydrogenase; AST: aspartate aminotransferase; ALT: alanine aminotransferase. All variables are means ± SD, except the variables CRP, LDH, lymphocytes, D-dimer, AST, and ALT, which are medians and IQR.

**Table 2:** Clinical and epidemiological characteristics of overweight/obese and lean kidney transplant recipients.

Variables	BMI ≥ 25 (*n* = 185, 65.1%)	BMI < 25 (*n* = 99, 34.9%)	TOTAL (*n* = 284, 100%)	Univariate Analysis
Age (years)	53.3 ± 11.1	50.9 ± 14.0	52.5 ± 12.2	1.02 (1.00–1.04, *p* = 0.12)
Male (*n*, %)	97 (60.6)	63 (39.4)	160 (56.3)	0.63 (0.38–1.04, *p* = 0.07)
Female (*n*, %)	88 (71)	36 (29)	124 (43.7)	1.59 (0.96–2.62, *p* = 0.07)
Race (*n*, %) White Black/brown	121 (65.4) 64 (34.6)	52 (52.5) 47 (47.5)	173 (60.9) 111 (39.1)	1.71 (1.04–2.81, *p* = 0.03)
Transplant time (months)	71.0 [33.0;145.0]	74.0 [34.5;127.5]	73.5 [33.0;142.3]	0.99 (1.00–1.00, *p* = 0.72)
Donor type (*n*, %) Alive Deceased	55 (29.7) 130 (70.3)	25 (25.3) 74 (74.7)	80 (28.2) 204 (71.8)	1.25 (0.72–2.17, *p* = 0.42)
BMI (kg/m^2^)	28.9 [26.0;31.2]	22.4 [20.2;23.6]	26.6 [23.5;29.6]	*p* = 0.92
Hypertension (*n*, %)	145 (78.4)	69 (69.7)	214 (75.4)	1.58 (0.91–2.74, *p* = 0.11)
Diabetes mellitus (*n*, %)	76 (41.1)	36 (36.4)	112 (39.4)	1.22 (0.74–2.02, *p* = 0.44)
COPD (*n*, %)	6 (3.2)	3 (3.0)	9 (3.2)	1.07 (0.26–4.38, *p* = 0.92)
Heart disease (*n*, %)	24 (13.0)	8 (8.1)	32 (11.3)	1.70 (0.73–3.93, *p* = 0.22)
Neoplasia (*n*, %)	13 (7.0)	8 (8.1)	21 (7.4)	0.86 (0.34–2.15, *p* = 0.75)
Liver disease (*n*, %)	6 (3.2)	3 (3.0)	9 (3.2)	1.07 (0.26–4.38, *p* = 0.92)
Autoimmune disease (*n*, %)	7 (3.8)	1 (1.0)	8 (2.8)	3.85 (0.47–31.78, *p* = 0.21)
Smoking (*n*, %)	43 (23.2)	16 (16.2)	59 (20.8)	1.72 (0.89–3.29, *p* = 0.10)

BMI: body mass index in kg/m^2^; COPD: chronic obstructive pulmonary disease. All variables are means ± SD, except transplant time and BMI, which are medians and IQR.

**Table 3:** Laboratory data and outcomes of overweight/obese and lean kidney transplant recipients.

Laboratory Data	BMI ≥ 25 (*n* = 185, 65.1%)	BMI < 25 (*n* = 99, 34.9%)	TOTAL (*n* = 291, 100%)	Univariate Analysis
Basal eGFR	49.2 ± 23.2	47.8 ± 25.6	48.7 ± 24.0	1.00 (0.99–1.01, *p* = 0.63)
Admission eGFR	37.7 ± 20.7	36.0 ± 24.0	37.1 ± 21.9	1.00 (0.99–1.01, *p* = 0.53)
Previous glucose (mg/dL)	125.5 ± 65.5	110.5 ± 67.5	120.3 ± 66.5	1.00 (1.00–1.01, *p* = 0.08)
Admission glucose (mg/dL)	184.9 ± 107.7	154.6 ± 95.9	174.8 ± 104.5	1.00 (1.00–1.01, *p* = 0.14)
Previous Hb1Ac (%)	7.0 ± 2.0	6.5 ± 2.0	6.9 ± 2.0	1.15 (0.98–1.34, *p* = 0.08)
CRP (mg/dL)	5.5 [2.0;13.0]	6.4 [1.8;11.3]	5.7 [2.0;12.7]	1.01 (0.98–1.04, *p* = 0.59)
LDH (U/L)	292.0 [224.0;407.0]	273.0 [212.5;370.3]	288.0 [220.0;395.0]	1.00 (1.00–1.00, *p* = 0.31)
Lymphocytes (mm³)	743.5 [498.0;1234.3]	762.5 [474.3;1127.5]	750.5 [497.0;1202.5]	1.00 (1.00–1.00, *p* = 0.87)
D-dimer (µg/L)	1.2 [0.6;2.3]	1.2 [0.6;2.3]	1.2 [0.6;2.3]	0.96 (0.89–1.03, *p* = 0.23)
AST (U/L)	28.5 [20.8;40.3]	28.0 [23.0;42]	28.0 [21.0;41.0]	1.00 (0.99–1.01, *p* = 0.78)
ALT (U/L)	21.0 [15.0;33.0]	21.0 [15.0;29.0]	21.0 [15.0;32.0]	1.01 (0.99--1.02, *p* = 0.29)
Sodium (mEq/L)	135.1 ± 4.9	134.6 ± 5.3	135.0 ± 5.1	1.11 (0.97–1.07, *p* = 0.48)
Outcomes				
Death (*n*, %)	59 (31.9)	25 (25.3)	84 (29.6)	1.39 (0.80–2.40, *p* = 0.24)
ICU (*n*, %)	92 (49.7)	42 (42.4)	134 (47.2)	1.34 (0.82–2.20, *p* = 0.24)
O_2_ (*n*, %)	108 (58.4)	45 (45.5)	153 (53.9)	1.69 (1.03–2.75, *p* = 0.04)
IMV (*n*, %)	70 (37.8)	27 (27.3)	97 (34.2)	1.62 (0.95–2.77, *p* = 0.07)
AKI (*n*, %)	103 (55.7)	62 (62.6)	165 (58.1)	0.75 (0.45–1.24, *p* = 0.26)
Stage 1	21 (11.4)	16 (16.2)	37 (13.0)	0.66 (0.33–1.34, *p* = 0.25)
Stage 2	9 (4.9)	5 (5.1)	14 (4.9)	0.96 (0.31–2.95, *p* = 0.94)
Stage 3	73 (39.4)	41 (41.4)	114 (40.1)	0.92 (0.56–0.51, *p* = 0.75)
HD (*n*, %)	70 (37.8)	35 (35.4)	105 (37.0)	1.11 (0.67–1.85, *p* = 0.68)

BMI: body mass index in kg/m^2^; eGFR: estimated glomerular rate, in mL/min/1.73 m^2^; Hb1Ac: glycated hemoglobin; CRP: C-reactive protein; LDH: lactate dehydrogenase; AST: aspartate aminotransferase; ALT: alanine aminotransferase. All variables are means ± SD, except the variables CRP, LDH, lymphocytes, D-dimer, AST, and ALT, which are medians and IQR.

**Table 4:** Risk factors for the use of supplemental oxygen (O_2_) in kidney transplant recipients who were overweight/obese.

Variables	O_2_ (*n* = 108, 58.4%)	No O_2_ (*n* = 77, 41.6%)	Univariate Analysis	Multivariate Analysis
Age (years)	55.7 ± 10.6	49.9 ± 10.8	1.05 (1.02–1.08, *p* = 0.001)	1.06 (1.03–1.11, *p* = 0.001)
Male (*n*, %)	52 (48.1)	45 (58.4)	0.67 (0.37–1.19, *p* = 0.17)	
Race (*n*, %) White Black/brown	71 (65.7) 37 (34.3)	50 (64.9) 27 (35.1)	1.04 (0.56–1.91, *p* = 0.91)	
Transplant time (months)	90.8 ± 73.4	94.1 ± 69.9	0.10 (1.00–0.99, *p* = 0.76)	
Donor type (*n*, %) Alive Deceased	30 (27.8) 78 (72.2)	25 (32.5) 52 (67.5)	0.80 (0.42–1.51, *p* = 0.49)	
Hypertension (*n*, %)	84 (77.8)	61 (79.2)	0.92 (0.45–1.87, *p* = 0.81)	
Diabetes mellitus (*n*, %)	50 (46.3)	26 (33.8)	1.69 (0.92–3.10, *p* = 0.06)	0.94 (0.44–2.03, *p* = 0.88)
COPD (*n*, %)	4 (3.7)	2 (2.6)	1.44 (0.26–8.08, *p* = 0.68)	
Heart disease (*n*, %)	14 (13.0)	10 (13.0)	0.99 (0.42–2.38, *p* = 0.10)	
Neoplasia (*n*, %)	8 (8.4)	5 (6.5)	1.15 (0.36–3.67, *p* = 0.81)	
Liver disease (*n*, %)	6 (5.6)	0 (0.0)	−(0.0001, *p* = 0.999)	
Autoimmune disease (*n*, %)	5 (4.6)	2 (2.6)	1.82 (0.34–9.37, *p* = 0.48)	
Smoking (*n*, %)	32 (29.6)	11 (14.3)	2.45 (2.45–1.12, *p* = 0.02)	2.07 (0.91–4.72, *p* = 0.08)
Laboratory data				
Basal eGFR	47.5 ± 22.0	51.6 ± 24.8	0.99 (0.98–1.00, *p* = 0.24)	
Admission eGFR	34.3 ± 20.3	42.5 ± 20.4	0.98 (0.97–0.99, *p* = 0.01)	0.98 (0.96–1.00, *p* = 0.08)
Previous glucose (mg/dL)	133.4 ± 73.2	114.5 ± 51.4	1.01 (1.00–1.01, *p* = 0.06)	0.10 (1.00–1.01, *p* = 0.54)
Admission glucose (mg/dL)	186.3 ± 107.9	181.0 ± 109.9	1.00 (1.00–1.00, *p* = 0.85)	
Previous Hb1Ac (%)	7.2 ± 2.1	6.8 ± 1.9	1.13 (0.945–1.357, *p* = 0.18)	
CRP (mg/dL)	7.4 [3.6;13.2]	3.4 [1.3;9.7]	1.03 (0.992–1.069, *p* = 0.12)	
LDH (U/L)	338.5 [240.8;432.0]	250.0 [196.0;342.0]	1.00 (1.00–1.00, *p* = 0.049)	1.03 (0.98–1.09, *p* = 0.24)
Lymphocytes (mm^3^)	674.0 [465.3;1087.3]	900.5 [597.3;1533.0]	0.10 (1.00–1.00, *p* = 0.003)	1.00 (1.00–1.00, *p* = 0.40)
D-dimer (µg/L)	1.3 [0.6;2.3]	1.1 [0.6;2.1]	1.04 (0.93–1.16, *p* = 0.52)	
AST (U/L)	27.5 [19.3;41.0]	29.0 [22.3;37.8]	1.01 (1.00–1.01, *p* = 0.36)	
ALT (U/L)	20.5 [12.3;33.0]	22.0 [16.0;32.0]	1.00 (1.00–1.01, *p* = 0.38)	
Sodium (mEq/L)	134.5 ± 5.6	136.1 ± 3.4	0.92 (0.85–1.00, *p* = 0.04)	0.93 (0.84–1.03, *p* = 0.18)

BMI: body mass index in kg/m^2^; COPD: chronic obstructive pulmonary disease; eGFR in mL/min/1.73 m^2^; Hb1Ac: glycated hemoglobin; CRP: C-reactive protein; LDH: lactate dehydrogenase; AST: aspartate aminotransferase; ALT: alanine aminotransferase. All variables are means ± SD, except the variables transplant time, CRP, LDH, lymphocytes, D-dimer, AST, and ALT, which are medians and IQR.

## Data Availability

The data presented in this study are available on request from the corresponding authors. The data are not publicly available due to clinical patient information.

## Supplementary Materials

The following supporting information can be downloaded at https://www.mdpi.com/article/10.3390/diagnostics13132168/s1, Table S1: Risk factors for mortality in kidney transplant recipients. Table S2: Risk factors for intensive care unit (ICU) admission in kidney transplant recipients. Table S3: Risk factors for the need for invasive mechanical ventilation (IMV) admission in kidney transplant recipients. Table S4: Risk factors for acute kidney injury (AKI) in kidney transplant recipients. Table S5: Risk factors for the need for hemodialysis (HD) admission in kidney transplant recipients. Table S6: Clinical and epidemiological characteristics of overweight/obese and lean kidney transplant recipients (*n* = 284) using BMI as a continuous variable. Table S7: Laboratory data and outcomes of overweight/obese and lean KTRs (*n* = 284) using BMI as a continuous variable.

## References

[1] Kates O.S., Haydel B.M., Florman S.S., Rana M.M., Chaudhry Z.S., Ramesh M.S., Safa K., Kotton C.N., Blumberg E.A., Besharatian B.D. (2021). Coronavirus Disease 2019 in Solid Organ Transplant: A Multicenter Cohort Study. Clin. Infect. Dis..

[2] Chavarot N., Gueguen J., Bonnet G., Jdidou M., Trimaille A., Burger C., Amrouche L., Weizman O., Pommier T., Aubert O. (2021). COVID-19 severity in kidney transplant recipients is similar to nontransplant patients with similar comorbidities. Am. J. Transplant..

[3] Aguiar-Brito I., de Lucena D.D., Veronese-Araujo A., Cristelli M.P., Tedesco-Silva H., Medina-Pestana J.O., Rangel E.B. (2022). Impact of Hypertension on COVID-19 Burden in Kidney Transplant Recipients: An Observational Cohort Study. Viruses.

[4] Fisher A.M., Schlauch D., Mulloy M., Dao A., Reyad A.I., Correll M., Fromell G.J., Pittman J., Bingaman A.W., Sankarapandian B. (2021). Outcomes of COVID-19 in hospitalized solid organ transplant recipients compared to a matched cohort of non-transplant patients at a national healthcare system in the United States. Clin. Transplant..

[5] Linares L., Cofan F., Diekmann F., Herrera S., Marcos M.A., Castel M.A., Farrero M., Colmenero J., Ruiz P., Crespo G. (2021). A propensity score-matched analysis of mortality in solid organ transplant patients with COVID-19 compared to non-solid organ transplant patients. PLoS ONE.

[6] Rangel E.B., de Lucena D.D., Aguiar-Brito I., de Andrade L.G.M., Veronese-Araujo A., Cristelli M.P., Tedesco-Silva H., Medina-Pestana J.O. (2022). COVID-19 in Kidney Transplant Recipients with Diabetes Mellitus: A Propensity Score Matching Analysis. Transpl. Int..

[7] Yang J., Hu J., Zhu C. (2021). Obesity aggravates COVID-19: A systematic review and meta-analysis. J. Med. Virol..

[8] Lasbleiz A., Gaborit B., Soghomonian A., Bartoli A., Ancel P., Jacquier A., Dutour A. (2021). COVID-19 and Obesity: Role of Ectopic Visceral and Epicardial Adipose Tissues in Myocardial Injury. Front. Endocrinol..

[9] De Freitas V.M., Chiloff D.M., Bosso G.G., Teixeira J.O.P., Hernandes I.C.G., Padilha M.D.P., Moura G.C., de Andrade L.G.M., Mancuso F., Finamor F.E. (2022). A Machine Learning Model for Predicting Hospitalization in Patients with Respiratory Symptoms during the COVID-19 Pandemic. J. Clin. Med..

[10] Marshall J.C., Murthy S., Diaz J., Adhikari N.K., Angus D.C., Arabi Y.M., Baillie K., Bauer M., Berry S., Blackwood B. (2020). A minimal common outcome measure set for COVID-19 clinical research. Lancet Infect. Dis..

[11] Saadatmand S., Salimifard K., Mohammadi R., Marzban M., Naghibzadeh-Tahami A. (2022). Predicting the necessity of oxygen therapy in the early stage of COVID-19 using machine learning. Med. Biol. Eng. Comput..

[12] Stefan N., Birkenfeld A.L., Schulze M.B., Ludwig D.S. (2020). Obesity and impaired metabolic health in patients with COVID-19. Nat. Rev. Endocrinol..

[13] Shin J., Toyoda S., Nishitani S., Fukuhara A., Kita S., Otsuki M., Shimomura I. (2021). Possible Involvement of Adipose Tissue in Patients with Older Age, Obesity, and Diabetes with SARS-CoV-2 Infection (COVID-19) via GRP78 (BIP/HSPA5): Significance of Hyperinsulinemia Management in COVID-19. Diabetes.

[14] Beyerstedt S., Casaro E.B., Rangel E.B. (2021). COVID-19: Angiotensin-converting enzyme 2 (ACE2) expression and tissue susceptibility to SARS-CoV-2 infection. Eur. J. Clin. Microbiol. Infect. Dis..

[15] Al-Benna S. (2020). Association of high level gene expression of ACE2 in adipose tissue with mortality of COVID-19 infection in obese patients. Obes. Med..

[16] Li Y., Xu Q., Ma L., Wu D., Gao J., Chen G., Li H. (2020). Systematic profiling of ACE2 expression in diverse physiological and pathological conditions for COVID-19/SARS-CoV-2. J. Cell. Mol. Med..

[17] Kornilov S.A., Lucas I., Jade K., Dai C.L., Lovejoy J.C., Magis A.T. (2020). Plasma levels of soluble ACE2are associated with sex, Metabolic Syndrome, and its biomarkers in a large cohort, pointing to a possible mechanism for increased severity in COVID-19. Crit. Care.

[18] Higashikuni Y., Liu W., Obana T., Sata M. (2021). Pathogenic Basis of Thromboinflammation and Endothelial Injury in COVID-19: Current Findings and Therapeutic Implications. Int. J. Mol. Sci..

[19] Nagashima S., Mendes M.C., Camargo Martins A.P., Borges N.H., Godoy T.M., Miggiolaro A.F.R.D., da Silva D.F., Machado-Souza C., de Noronha L. (2020). Endothelial Dysfunction and Thrombosis in Patients with COVID-19-Brief Report. Arterioscler. Thromb. Vasc. Biol..

[20] Fuster J.J., Ouchi N., Gokce N., Walsh K. (2016). Obesity-Induced Changes in Adipose Tissue Microenvironment and Their Impact on Cardiovascular Disease. Circ. Res..

[21] Salvator H., Grassin-Delyle S., Naline E., Brollo M., Fournier C., Couderc L.J., Devillier P. (2020). Contrasting Effects of Adipokines on the Cytokine Production by Primary Human Bronchial Epithelial Cells: Inhibitory Effects of Adiponectin. Front. Pharmacol..

[22] Dana R., Bannay A., Bourst P., Ziegler C., Losser M.R., Gibot S., Levy B., Audibert G., Ziegler O. (2021). Obesity and mortality in critically ill COVID-19 patients with respiratory failure. Int. J. Obes..

[23] De J.A., Chanques G., Jaber S. (2017). Mechanical ventilation in obese ICU patients: From intubation to extubation. Crit. Care.

[24] Kress J.P., Pohlman A.S., Alverdy J., Hall J.B. (1999). The impact of morbid obesity on oxygen cost of breathing (VO(2RESP)) at rest. Am. J. Respir. Crit. Care Med..

[25] Chlif M., Keochkerian D., Choquet D., Vaidie A., Ahmaidi S. (2009). Effects of obesity on breathing pattern, ventilatory neural drive and mechanics. Respir. Physiol. Neurobiol..

[26] Pan X.W., Xu D., Zhang H., Zhou W., Wang L.H., Cui X.G. (2020). Identification of a potential mechanism of acute kidney injury during the COVID-19 outbreak: A study based on single-cell transcriptome analysis. Intensive Care Med..

[27] de Lucena D.D., Rangel E.B. (2018). Glucocorticoids use in kidney transplant setting. Expert Opin. Drug Metab. Toxicol..

[28] Pinto B.G.G., Oliveira A.E.R., Singh Y., Jimenez L., Goncalves A.N.A., Ogava R.L.T., Creighton R., Schatzmann Peron J.P., Nakaya H.I. (2020). ACE2 Expression Is Increased in the Lungs of Patients with Comorbidities Associated with Severe COVID-19. J. Infect. Dis..

[29] Antos A., Kwong M.L., Balmorez T., Villanueva A., Murakami S. (2021). Unusually High Risks of COVID-19 Mortality with Age-Related Comorbidities: An Adjusted Meta-Analysis Method to Improve the Risk Assessment of Mortality Using the Comorbid Mortality Data. Infect. Dis. Rep..

[30] Bartleson J.M., Radenkovic D., Covarrubias A.J., Furman D., Winer D.A., Verdin E. (2021). SARS-CoV-2, COVID-19 and the Ageing Immune System. Nat. Aging.

[31] Mahmoodpoor A., Hosseini M., Soltani-Zangbar S., Sanaie S., Aghebati-Maleki L., Saghaleini S.H., Ostadi Z., Hajivalili M., Bayatmakoo Z., Haji-Fatahaliha M. (2021). Reduction and exhausted features of T lymphocytes under serological changes, and prognostic factors in COVID-19 progression. Mol. Immunol..

[32] Cravedi P., Mothi S.S., Azzi Y., Haverly M., Farouk S.S., Perez-Saez M.J., Redondo-Pachon M.D., Murphy B., Florman S., Cyrino L.G. (2020). COVID-19 and kidney transplantation: Results from the TANGO International Transplant Consortium. Am. J. Transplant..

[33] Fava A., Cucchiari D., Montero N., Toapanta N., Centellas F.J., Vila-Santandreu A., Coloma A., Meneghini M., Manonelles A., Sellares J. (2020). Clinical characteristics and risk factors for severe COVID-19 in hospitalized kidney transplant recipients: A multicentric cohort study. Am. J. Transplant..

[34] Martha J.W., Wibowo A., Pranata R. (2022). Prognostic value of elevated lactate dehydrogenase in patients with COVID-19: A systematic review and meta-analysis. Postgrad. Med. J..

[35] Gansevoort R.T., Hilbrands L.B. (2020). CKD is a key risk factor for COVID-19 mortality. Nat. Rev. Nephrol..

[36] Cheng Y., Luo R., Wang K., Zhang M., Wang Z., Dong L., Li J., Yao Y., Ge S., Xu G. (2020). Kidney disease is associated with in-hospital death of patients with COVID-19. Kidney Int..

[37] Hirsch J.S., Ng J.H., Ross D.W., Sharma P., Shah H.H., Barnett R.L., Hazzan A.D., Fishbane S., Jhaveri K.D. (2020). Acute kidney injury in patients hospitalized with COVID-19. Kidney Int..

[38] Gheorghe G., Ilie M., Bungau S., Stoian A.M.P., Bacalbasa N., Diaconu C.C. (2021). Is There a Relationship between COVID-19 and Hyponatremia?. Medicina.

[39] Akbar M.R., Pranata R., Wibowo A., Irvan, Sihite T.A., Martha J.W. (2021). The Prognostic Value of Hyponatremia for Predicting Poor Outcome in Patients with COVID-19: A Systematic Review and Meta-Analysis. Front. Med..

[40] De Lucena D.D., de Sa J.R., Medina-Pestana J.O., Rangel E.B. (2020). Modifiable Variables Are Major Risk Factors for Posttransplant Diabetes Mellitus in a Time-Dependent Manner in Kidney Transplant: An Observational Cohort Study. J. Diabetes Res..

