# Supplementary material for: Oxygen Requirement in Overweight/Obese Kidney Transplant Recipients with COVID-19: An Observational Cohort Study
Source: Diagnostics (Basel). 2023 Jun 26;13(13):2168. doi: 10.3390/diagnostics13132168 (PMC10340440; doi:10.3390/diagnostics13132168)
Supplement: Supplementary file 1 [file diagnostics-13-02168-s001.zip › diagnostics-2352795-supplementary.pdf]

Supplementary file

Tables S1–S5 denote univariate analyses and multivariate binary logistic regression models.
BMI: body mass index in kg/m^2; eGFR: estimated glomerular rate, in mL/min/1.73 m²; Hb1Ac: glycated hemoglobin; CRP: C-reactive protein; LDH: lactate dehydrogenase; AST: aspartate aminotransferase; ALT: alanine aminotransferase; ICU: intensive care unit; O_2: use of supplemental oxygen; IMV: invasive mechanical ventilation; AKI: acute kidney injury; HD: hemodialysis. All variables are means ± SD, except the variables CRP, LDH, lymphocytes, D-dimer, AST, and ALT, which are medians and IQR.

Table S1. Risk factors for mortality in kidney transplant recipients.

**Variables**	**NOT ALIVE (n=84, 29.6%)**	**ALIVE (n=200, 70.4%)**	**Univariate analysis**	**Multivariate analysis**
Age (years)	58.6 ± 11.0	49.9 ± 11.8	1.07 (1.04-1.09, ***p=0.0001***)	1.05 (1.02-1.08, ***p=0.001***)
Male (n, %)	52 (61.9)	108 (54.0)	1.38 (0.82-2.33, p=0.22)	
Race (n, %)				
White	51 (60.7)	122 (61.0)	0.99 (0.59-1.17, p=0.96)	
Black/brown	33 (39.3)	78 (39.0)		
Transplant time (months)	95.0 ± 70.9	92.5 ± 72.3	1.00 (1.00-1.00, p=0.79)	
Donor type (n, %)				
Live	17 (20.2)	63 (31.5)	1.81 (0.98-3.34, p=0.056)	1.09 (0.48-2.49, p=0.84)
Deceased	67 (79.8)	137 (68.5)		
BMI (kg/m²)	27.1 ± 4.7	26.8 ± 5.0	1.01 (0.96-1.06, p=0.72)	
BMI ≥ 25 (n, %)	59 (70.2)	126 (63.0)	1.39 (0.80-2.40, p=0.24)	
BMI ≥ 30 (n, %)	21 (25.0)	44 (22.0)	1.18 (0.65-2.15, p=0.58)	
Hypertension (n, %)	69 (82.1)	145 (72.5)	1.75 (0.92-3.30, p=0.089)	1.55 (0.70-3.40, p=0.28)
Diabetes mellitus (n, %)	43 (51.2)	69 (34.5)	1.99 (1.19-3.34, ***p=0.009***)	1.07 (0.54-2.09, p=0.85)
COPD (n, %)	3 (3.6)	6 (3.0)	1.20 (0.29-4.90, p=0.80)	
Heart disease (n, %)	18 (21.4)	14 (7.0)	3.62 (1.71-7.69, ***p=0.001***)	3.20 (1.20-8.57, ***p=0.02***)
Neoplasia (n, %)	10 (11.9)	11 (5.5)	2.32 (0.95-5.70, p=0.07)	1.96 (0.51-7.52, p=0.32)
Liver disease (n, %)	5 (6.0)	4 (2.0)	3.10 (0.81-11.85, p=0.10)	0.90 (0.18-4.61, p=0.90)
Autoimmune disease (n, %)	3 (3.6)	5 (2.5)	1.44 (0.34-6.19, p=0.62)	
Smoking (n, %)	25 (29.8)	34 (17)	2.08 (1.12-3.85, ***p=0.02***)	1.890 (0.96-3.70, p=0.06)
**Laboratory data**				
Basal eGFR	47.3 ± 26.0	49.3 ± 23.2	1.0 (1.00-1.00, p=0.51)	

Admission eGFR	33.5 ± 22.3	38.6 ± 21.6	0.99 (1.00-1.00, p=0.08)	0.99 (0.96-1.01, p=0.30)
Previous glucose (mg/dL)	147.3 ± 81.2	108.9 ± 55.7	1.01 (1.00-1.01, ***p=0.0001***)	1.00 (0.99-1.01, p=0.62)
Admission glucose (mg/dL)	193.7 ± 112.0	158.8 ± 95.8	1.00 (1.00-1.00, p=0.07)	1.00 (1.00-1.02, p=0.23)
Previous Hb1Ac (%)	7.3 ± 2.3	6.7 ± 1.9	1.15 (1.01-1.32, ***p=0.04***)	0.66 (0.30-1.43, p=0.29)
CRP (mg/dL)	11.1 [5.5;17.2]	4.4 [1.4;10.3]	1.08 (1.04-1.11, ***p=0.0001***)	0.96 (0.87-1.06, p=0.47)
LDH (U/L)	359.0 [286.0;441.0]	266.0 [208.0;353.3]	1.00 (1.00-1.00, ***p=0.02***)	1.00 (1.00-1.00, p=0.37)
Lymphocytes (mm^3)	549.5 [368.3;849.0]	860.5 [583.5;1218.8]	1.00 (1.00-1.00, ***p=0.02***)	1.00 (1.00-1.00, p=0.12)
D-dimer (µg/L)	1.4 [0.7;2.8]	1.1 [0.5;2.1]	1.12 (1.04-1.21, ***p=0.003***)	1.47 (0.95-2.27, p=0.08)
AST (U/L)	34.0 [15.0;33.0]	28.0 [21.0;40.0]	1.00 (1.00-1.01, p=0.43)	
ALT (U/L)	24.0 [21.0;43.3]	21.0 [15.0;30.5]	1.00 (1.00-1.01, p=0.33)	
Sodium (mEq/L)	134.6 ± 5.9	135.1 ± 4.7	0.98 (0.93-1.04, p=0.48)	

Table S2. Risk factors for intensive care unit (ICU) admission in kidney transplant recipients.

Variables	ICU (n=134, %)	No ICU (n=150, %)	Univariate analysis	Multivariate analysis
Age (years)	56.2 ± 11.7	49.2 ± 11.7	1.05 (1.03-1.07, ***p=0.0001***)	1.04 (1.02-1.07, ***p=0.0001***)
Male (n, %)	76 (56.7)	84 (56.0)	1.03 (0.64-1.65, p=0.90)	
Race (n, %)				
White	83 (61.9)	90 (60)	1.09 (0.67-1.75, p=0.74)	
Black/brown	51 (38.1)	60 (40)		
Transplant time (months)	92.6 ± 70.1	93.8 ± 73.5	1.00 (1.00-1.00, p=0.90)	
Donor type (n, %)				
Live	37 (27.6)	43 (28.7)	0.95 (0.56-1.59, p=0.84)	
Deceased	97 (72.4)	107 (71.3)		
BMI (kg/m²)	27.3 ± 4.8	26.5 ± 4.9	1.04 (1.00-1.09, p=0.16)	
BMI ≥ 25 (n, %)	92 (68.7)	93 (62.0)	1.34 (0.82-2.20, p=24)	
BMI ≥ 30 (n, %)	35 (26.1)	30 (20.0)	1.41 (0.81-2.46, p=0.22)	
Hypertension (n, %)	108 (80.6)	106 (70.7)	1.72 (0.99-3.00, p=0.05)	1.49 (0.82-2.71, p=0.19)
Diabetes mellitus (n, %)	60 (44.8)	52 (34.7)	1.53 (0.95-2.50, p=0.08)	1.00 (0.58-1.71, p=0.99)
COPD (n, %)	6 (4.5)	3 (2.0)	2.30 (0.56-9.37, p=0.25)	
Heart disease (n, %)	24 (17.9)	8 (5.3)	3.87 (1.67-8.95, ***p=0.002***)	2.31 (0.95-5.62, p=0.06)
Neoplasia (n, %)	15 (11.2)	6 (4.0)	3.03 (1.14-8.04, ***p=0.03***)	1.86 (0.64-5.40, p=0.25)
Liver disease (n, %)	6 (4.5)	3 (2.0)	2.30 (0.56-9.37, p=0.25)	
Autoimmune disease (n, %)	6 (4.5)	2 (1.3)	3.47 (0.69-17.49, p=0.13)	
Smoking (n, %)	34 (25.4)	25 (16.7)	1.56 (0.86-2.84, p=0.14)	
**Laboratory data**				
Basal eGFR	47.3 ± 26.3	50.0 ± 21.8	1.00 (0.99-1.00, p=0.35)	
Admission eGFR	33.2 ± 22.3	40.6 ± 21.0	0.98 (0.97-0.99, ***p=0.005***)	0.98 (0.95-1.00, p=0.07)
Previous glucose (mg/dL)	136.6 ± 71.5	105.7 ± 58.2	1.01 (1.00-1.01, ***p=0.0001***)	1.00 (0.99-1.00, p=0.57)
Admission glucose (mg/dL)	181.5 ± 107.4	161.6 ± 98.5	1.00 (1.00-1.00, p=0.33)	
Previous Hb1Ac (%)	7.2 ± 2.2	6.6 ± 1.8	1.17 (1.02-1.35, ***p=0.03***)	1.142 (0.87-1.49, p=33)
CRP (mg/dL)	9.3 [4.1;15.7]	3.7 [1.1;9.6]	1.07 (1.03-1.10, ***p=0.0001***)	0.98 (0.92-1.05, p=0.61)
LDH (U/L)	347.0 [242.0;467.0]	247.5 [202.0;309.3]	1.01 (1.00-1.00, ***p=0.0001***)	1.00 (1.00-1.00, ***p=0.03***)
Lymphocytes (mm³)	594.0 [378.3;915.8]	921.0 [683.5;1243.5]	1.00 (1.00-1.00, ***p=0.03***)	1.00 (1.00-1.00, p=0.07)
D-dimer (µg/L)	1.5 [0.8;2.8]	0.9 [0.5;1.7]	1.15 (1.04-1.27, ***p=0.005***)	1.19 (0.97-1.45, p=0.09)
AST (U/L)	31.5 [21.0;42.0]	28.0 [21.0;40.0]	1.01 (1.00-1.02, p=0.10)	
ALT (U/L)	22.0 [14.0;31.0]	21.0 [15.3;32.8]	1.00 (0.99-1.01, p=0.59)	

Sodium (mEq/L)	133.8 ± 6.1	136.0 ± 3.7	0.91 (0.86-0.97, ***p=0.002***)	0.90 (0.81-1.00, ***p=0.04***)

Table S3. Risk factors for the need for invasive mechanical ventilation (IMV) admission in kidney transplant recipients.

**Variables**	**IMV (n=97, 34.2%)**	**No IMV (n=187, 65.8%)**	**Univariate analysis**	**Multivariate analysis**
Age (years)	57.3 ± 10.7	50.0 ± 12.2	1.01 (1.03-1.08, ***p=0.0001***)	1.04 (1.01-1.06, ***p=0.006***)
Male (n, %)	61 (62.9)	99 (52.9)	1.51 (0.91-2.49, p=0.11)	
Race (n, %)			0.99 (0.60-1.64, p=0.98)	
White	59 (60.8)	114 (61.0)		
Black/brown	38 (39.2)	73 (39.0)		
Transplant time (months)	92.5 ± 69.0	93.6 ± 73.4	1.00 (1.00-1.00, p=0.90)	
Donor type (n, %)			1.53 (0.87-2.70, p=0.14)	
Live	22 (22.7)	58 (31.0)		
Deceased	75 (77.3)	129 (69.0)		
BMI (kg/m²)	27.5 ± 4.5	26.6 ± 5.0	1.04 (0.99-1.09, p=0.12)	
BMI ≥ 25 (n, %)	70 (72.2)	115 (61.5)	1.62 (0.95-2.77, p=0.07)	
BMI ≥ 30 (n, %)	26 (26.8)	39 (20.9)	1.39 (0.78-2.46, p=0.26)	
Hypertension (n, %)	79 (81.4)	135 (72.2)	1.69 (0.92-3.09, p=0.09)	1.54 (0.75-3.17, p=0.24)
Diabetes mellitus (n, %)	48 (49.5)	64 (34.2)	1.88 (1.14-3.10, ***p=0.01***)	1.17 (0.62-2.19, p=0.62)
COPD (n, %)	4 (4.1)	5 (2.7)	1.57 (0.41-5.97, p=0.51)	
Heart disease (n, %)	18 (18.6)	14 (7.5)	2.82 (1.33-5.94, ***p=0.007***)	2.30 (0.86-6.11, p=0.10)
Neoplasia (n, %)	12 (12.4)	9 (4.8)	2.79 (1.13-6.88, ***p=0.03***)	3.49 (0.90-13.52, p=0.07)

Liver disease (n, %)	6 (6.2)	3 (1.6)	4.04 (0.99-16.54, ***p=0.05***)	1.25 (0.24-6.44, p=0.79)
Autoimmune disease (n, %)	3 (3.1)	5 (2.7)	1.16 (0.27-4.97, p=0.84)	
Smoking (n, %)	27 (27.8)	32 (17.1)	1.75 (0.96-3.19, p=0.07)	1.60 (0.84-3.04, p=0.16)
**Laboratory data**				
Basal eGFR	48.1 ± 26.5	49.1 ± 22.8	1.00 (0.99-1.01, p=0.73)	
Admission eGFR	33.5 ± 22.3	39.0 ± 21.5	0.99 (0.98-1.00, ***p=0.048***)	0.97 (0.94-1.01, p=0.12)
Previous glucose (mg/dL)	144.9 ± 77.7	107.5 ± 55.9	1.01 (1.00-1.01, ***p=0.0001***)	0.99 (0.98-1.01, p=0.34)
Admission glucose (mg/dL)	199.5 ± 115.9	6.6 ± 1.8	1.01 (1.00-1.01, ***p=0.01***)	1.01 (1.00-1.02, p=0.19)
Previous Hb1Ac (%)	7.4 ± 2.3	150.2 ± 85.8	1.22 (1.06-1.40, ***p=0.006***)	1.27 (0.59-2.71, p=0.55)
CRP (mg/dL)	10.4 [4.9;16.8]	4.4 [1.4;10.3]	1.06 (1.02-1.09, ***p=0.001***)	0.95 (0.86-1.04, p=0.23)
LDH (U/L)	360.0 [263.0;486.5]	266.0 [209.5;347.0]	1.00 (1.00-1.00, ***p=0.006***)	1.00 (1.00-1.01, p=0.24)
Lymphocytes (mm³)	534.5 [357.5;838.0]	871.0 [602.3;1248.8]	1.00 (1.00-1.00, ***p=0.003***)	1.00 (1.00-1.00, ***p=0.03***)
D-dimer (µg/L)	1.3 [0.7;2.7]	1.1 [0.5;2.1]	1.09 (1.01-1.17, ***p=0.02***)	1.19 (0.97-1.46, p=0.10)
AST (U/L)	34.5 [21.3;42.0]	28.0 [21.0;40.0]	1.00 (0.99-1.01, p=0.56)	
ALT (U/L)	24.0 [15.5;33.0]	21.0 [15.0;29.5]	1.00 (1.00-1.01, p=0.38)	
Sodium (mEq/L)	134.2 ± 6.3	135.4 ± 4.3	0.96 (0.90-1.01, p=0.09)	1.02 (0.90-1.15, p=0.78)

Table S4. Risk factors for acute kidney injury (AKI) in kidney transplant recipients.

**Variables**	**AKI (n=165, 58.1%)**	**No AKI (n=119, 41.9%)**	**Univariate analysis**	**Multivariate analysis**
Age (years)	53.7 ± 12.8	50.7 ± 11.2	1.02 (1.00-1.04, ***p=0.04***)	1.02 (0.998-1.039, p=0.08)
Male (n, %)	91 (55.2)	69 (58.0)	0.89 (0.55-1.43, p=0.63)	
Race (n, %)				
White	99 (60.0)	74 (62.2)	0.91 (0.56-1.48, p=0.71)	
Black/brown	66 (40.0)	45 (37.8)		
Transplant time (months)	90.6 ± 71.5	96.8 ± 72.3	1.00 (1.00-1.00, p=0.47)	
Donor type (n, %)				
Live	41 (24.8)	39 (32.8)	1.47 (0.88-2.48, p=0.14)	
Deceased	124 (75.2)	80 (67.2)		
BMI (kg/m²)	26.6 ± 4.9	27.3 ± 4.9	0.97 (0.92-1.02, p=0.22)	
BMI ≥ 25 (n, %)	103 (62.4)	82 (68.9)	0.75 (0.45-1.24, p=0.26)	
BMI ≥ 30 (n, %)	39 (23.6)	26 (21.8)	1.11 (0.63-1.95, p=0.72)	
Hypertension (n, %)	126 (76.4)	88 (73.9)	1.14 (0.66-1.96, p=0.64)	
Diabetes mellitus (n, %)	69 (41.8)	43 (36.1)	1.27 (0.78-2.06, p=0.33)	
COPD (n, %)	6 (3.6)	3 (2.5)	1.46 (0.36-5.95, p=0.50)	
Heart disease (n, %)	21 (12.7)	11 (9.2)	1.43 (0.66-3.09, p=0.36)	
Neoplasia (n, %)	15 (9.1)	6 (5.0)	1.88 (0.71-5.01, p=0.20)	
Liver disease (n, %)	8 (4.8)	1 (0.8)	6.01 (0.74-48.73, p=0.09)	5.05 (0.615-41.393, p=0.13)
Autoimmune disease (n, %)	6 (3.6)	2 (1.7)	2.21 (0.44-11.13, p=0.34)	
Smoking (n, %)	41 (24.8)	18 (15.1)	1.68 (0.89-3.16, p=0.11)	
**Laboratory data**				
Basal eGFR	47.2 ± 26.0	50.8 ± 21.0	0.99 (0.98-1.00, p=0.22)	
Admission eGFR	29.2 ± 20.2	48.0 ± 19.4	0.96 (0.94-0.97, ***p=0.0001***)	0.94 (0.90-0.99, ***p=0.01***)
Previous glucose (mg/dL)	131.4 ± 79.3	104.9 ± 38.3	1.01 (1.00-1.01, ***p=0.002***)	1.03 (0.99-1.06, p=0.12)
Admission glucose (mg/dL)	185.6 ± 111.2	146.3 ± 79.0	1.00 (1.00-1.01, p=0.07)	1.02 (1.00-1.03, p=0.09)
Previous Hb1Ac (%)	7.1 ± 2.1	6.6 ± 1.9	1.14 (0.98-1.31, p=0.08)	0.50 (0.17-1.45, p=0.20)
CRP (mg/dL)	7.9 [3.4;15.3]	3.7 [1.0;9.4]	1.07 (1.03-1.11, ***p=0.0001***)	0.90 (0.80-1.02, p=0.09)
LDH (U/L)	315.0 [235.5;431.5]	257.0 [210.0;343.5]	1.00 (1.00-1.00, ***p=0.007***)	1.00 (1.00-1.01, p=0.29)
Lymphocytes (mm³)	633.5 [423.0;1056.5]	909.5 [632.0;1337.3]	1.00 (1.00-1.00, p=0.07)	1.00 (1.00-1.00, p=0.47)
D-dimer (µg/L)	1.4 [0.7;2.5]	0.9 [0.5;1.8]	1.07 (0.99-1.17, p=0.10)	1.42 (0.84-2.38, p=0.19)
AST (U/L)	31.0 [20.3;42.0]	28.0 [21.0;39.0]	1.01 (1.00-1.02, p=0.19)	
ALT (U/L)	22.0 [14.0;30.5]	21.0 [15.8;33.0]	1.00 (0.99-1.01, p=0.68)	

Sodium (mEq/L)	134.5 ± 5.6	135.6 ± 4.0	0.96 (0.90-1.01, p=0.10)

Table S5. Risk factors for the need for hemodialysis (HD) admission in kidney transplant recipients.

**Variables**	**HD (n=105, 37.0%)**	**No HD (n=179, 63.0%)**	**Univariate analysis**	**Multivariate analysis**
Age (years)	55.6 ± 12.0	50.7 ± 12.0	1.04 (1.01-1.06, ***p=0.001***)	1.03 (1.01-1.05, ***p=0.01***)
Male (n, %)	64 (61.0)	96 (53.6)	1.35 (0.83-2.20, p=0.23)	
Race (n, %)				
White	66 (62.9)	107 (59.8)	1.14 (0.69-1.87, p=0.61)	
Black/brown	39 (37.7)	72 (40.2)		
Transplant time (months)	91.7 ± 69.7	94.1 ± 73.1	1.00 (1.00-1.00, p=0.78)	
Donor type (n, %)				
Live	23 (21.9)	57 (31.8)	1.67 (0.95-2.91, p=0.07)	1.51 (0.84-2.70, p=0.17)
Deceased	82 (78.1)	122 (68.2)		
BMI (kg/m²)	27.1 ± 4.6	26.8 ± 5.1	1.01 (0.96-1.06, p=0.59)	
BMI ≥ 25 (n, %)	70 (66.7)	115 (64.2)	1.11 (0.67-1.85, p=0.68)	
BMI ≥ 30 (n, %)	27 (25.7)	38 (21.2)	1.28 (0.73-2.26, p=0.39)	
Hypertension (n, %)	86 (81.9)	128 (71.5)	1.80 (1.00-3.26, p=0.05)	1.54 (0.82-2.88, p=0.17)
Diabetes mellitus (n, %)	48 (45.7)	64 (35.8)	1.51 (0.93-2.47, p=0.10)	1.03 (0.60-1.76, p=0.92)
COPD (n, %)	5 (4.8)	4 (2.2)	2.19 (0.57-8.33, p=0.25)	
Heart disease (n, %)	18 (17.1)	14 (7.8)	2.44 (1.16-5.14, ***p=0.020***)	1.73 (0.79-3.77, p=0.17)
Neoplasia (n, %)	11 (10.5)	10 (5.6)	1.98 (0.81-4.83, p=0.13)	
Liver disease (n, %)	7 (6.7)	2 (1.1)	6.32 (1.29-31.02, ***p=0.02***)	
Autoimmune disease (n, %)	4 (3.8)	4 (2.2)	1.73 (0.42-7.08, p=0.44)	
Smoking (n, %)	28 (26.7)	31 (17.3)	1.52 (0.84-2.77, p=0.17)	
**Laboratory data**				
Basal eGFR	43.8 ± 26.1	51.6 ± 22.3	0.99 (0.97-1.00, ***p=0.01***)	1.01 (0.97-1.04, p=0.60)
Admission eGFR	28.9 ± 21.2	41.9 ± 20.9	0.97 (0.95-0.98, ***p=0.0001***)	0.96 (0.91-1.00, ***p=0.04***)
Previous glucose (mg/dL)	139.9 ± 76.8	108.7 ± 56.7	1.01 (1.00-1.01, ***p=0.0001***)	0.99 (0.97-1.01, p=0.250)
Admission glucose (mg/dL)	191.8 ± 115.4	156.0 ± 88.3	1.00 (1.00-1.00, p=0.07)	1.01 (0.99-1.01, p=0.32)
Previous Hb1Ac (%)	7.3 ± 2.3	6.6 ± 1.8	1.17 (1.02-1.34, ***p=0.03***)	1.15 (0.57-2.31, p=0.70)
CRP (mg/dL)	10.1 [4.3;15.8]	4.0 [1.4;10.3]	1.05 (1.02-1.08, ***p=0.003***)	0.94 (0.86-1.03, p=0.22)
LDH (U/L)	356.0 [240.0;486.5]	266.0 [210.0;346.3]	1.00 (1.00-1.00, ***p=0.002***)	1.00 (1.00-1.01, p=0.250)
Lymphocytes (mm³)	549.5 [378.8;843.0]	874.0 [604.8;1257.3]	1.00 (1.00-1.00, ***p=0.04***)	1.00 (1.00-1.00, p=0.14)
D-dimer (µg/L)	1.4 [0.7;2.6]	1.1 [0.5;2.1]	1.07 (0.99-1.15, p=0.07)	1.12 (0.94-1.42, p=0.17)
AST (U/L)	30.0 [20.0;42.0]	28.0 [22.0;40.0]	1.00 (0.99-1.01, p=0.72)	
ALT (U/L)	23.0 [14.0;32.0]	21.0 [15.0;31.8]	1.00 (1.00-1.01, p=0.50)	

Sodium (mEq/L)	134.1 ± 6.0	135.4 ± 4.3	0.95 (0.902-1.003, p=0.06)	1.00 (0.885-1.121, p=0.95)

Tables S6–S7 denote univariate analyzes and linear regression models using BMI as a continuous variable.

Table S6. Clinical and epidemiological characteristics of overweight/obese and lean kidney transplant recipients (n=284) using BMI as a continuous variable.

**Variables**	**Univariate Analysis**
Age (years)	0.02 (-0.025-0.068, p=0.36)
Male (n, %)	-0.73 (-1.881-0.415, p=0.21)
Female (n, %)	0.73 (-0.415-1.881, p=0.21)
Race (n, %)	
White	0.74 (-0.426-1.908, p=0.21)
Black/brown	
Transplant time (months)	0.00 (-0.007-0.009, p=0.88)
Donor type (n, %)	
Live	0.88 (-0.387-2.143, p=0.17)
Deceased	
Hypertension (n, %)	1.15 (-0.172-2.464, p=0.09)
Diabetes mellitus (n, %)	0.76 (-0.401-1.929, p=0.20)
COPD (n, %)	-1.60 (-4.856-1.653, p=0.33)
Heart disease (n, %)	1.07 (-0.736-2.867, p=0.24)

Neoplasia (n, %)	-0.19 (-2.368-1.996, p=0.87)
Liver disease (n, %)	-0.91 (-4.163-2.353, p=0.58)
Autoimmune disease (n, %)	-0.06 (-3.507-3.396, p=0.97)
Smoking (n, %)	-0.09 (-1.562-1.384, p=0.90)

BMI: body mass index in kg/m^2; COPD: chronic obstructive pulmonary disease.

Table S7. Laboratory data and outcomes of overweight/obese and lean KTRs (n=284) using BMI as a continuous variable.

**Laboratory data**	**Univariate Analysis**	**Multivariate Analysis**
Basal eGFR	0.02 (-0.008-0.039, p=0.20)	
Admission eGFR	0.02 (-0.004-0.048, p=0.09)	0.00 (-0.048-0.054, p=0.90)
Previous glucose (mg/dL)	0.01 (0.001-0.018, **p=0.04**)	-0.01 (-0.025-0.012, p=0.50)
Admission glucose (mg/dL)	0.01 (0.0001-0.018, p=0.05)	0.01 (-0.010-0.022, p=0.46)
Previous Hb1Ac (%)	0.42 (0.085-0.757, **p=0.01**)	0.42 (-0.570-1.406, p=0.40)
CRP (mg/dL)	0.01 (-0.067-0.077, p=0.88)	
LDH (U/L)	0.00 (0.0001-0.007, p=0.08)	0.01 (-0.001-0.013, p=0.08)
Lymphocytes (mm^3)	0.00 (0.0001-0.001, p=0.46)	
D-dimer (µg/L)	-0.07 (-0.245-0.102, p=0.42)	
AST (U/L)	0.01 (-0.003-0.030, p=0.10)	
ALT (U/L)	0.01 (-0.004-0.024, p=0.16)	
Sodium (mEq/L)	0.78 (-0.045-0.198, p=0.22)	
**Outcomes**		
Death (n, %)	0.23 (-1.025-1.477, p=0.72)	
ICU (n, %)	0.82 (-0.316-1.964, p=0.16)	
O_2 (n, %)	1.27 (0.132-2.403, **p=0.03**)	
IMV (n, %)	0.95 (-0.248-2.150, p=0.12)	
AKI (n, %)	-0.72 (-1.876-0.433, p=0.22)	
Stage 1	-0.60 (-2.296-1.094, p=0.49)	
Stage 2	-1.86 (-4.488-0.769, p=0.16)	
Stage 3	-0.09 (-1.250-1.080, p=0.91)	
HD (n, %)	0.32 (-0.858-1.506, p=0.59)	

BMI: body mass index in kg/m^2; eGFR: estimated glomerular rate, in mL/min/1.73 m^2; Hb1Ac: glycated hemoglobin; CRP: C-reactive protein; LDH: lactate dehydrogenase; AST: aspartate aminotransferase; ALT: alanine aminotransferase; ICU: intensive care unit; O_2: use of supplemental oxygen; IMV: invasive mechanical ventilation; AKI: acute kidney injury; HD: hemodialysis.
